# Elemi essential oil nanocapsulated drug ameliorates lung cancer via oxidative stress, apoptosis and inflammation pathway

**DOI:** 10.1111/jcmm.17801

**Published:** 2023-06-07

**Authors:** Beril Kurban, Tuğba Tuncel, Şennur Görgülü, Fatih Kar, Alper Öztürk, Temel Özek

**Affiliations:** ^1^ Faculty of Pharmacy Anadolu University Eskişehir Turkey; ^2^ Department of Pharmacognosy, Faculty of Pharmacy Anadolu University Eskişehir Turkey; ^3^ Medicinal Plant, Drug and Scientific Reasearch and Application Center (AUBİBAM) Anadolu University Eskişehir Turkey; ^4^ Department of Biochemistry, Faculty of Medicine Kütahya Health Sciences University Kütahya Turkey; ^5^ Department of Pharmaceutical Technology, Faculty of Pharmacy Anadolu University Eskişehir Turkey

**Keywords:** A549, CCD‐19Lu, Elemi oil, MTT, nanoparticle

## Abstract

Lung cancer is one of the most common causes of death in the world. Considering the severe side effects, toxicity and high costs of chemotherapeutics used in cancer treatment, there is a need for more economical and natural treatment methods such as essential oils. The purpose of this study is to determine the efficacy of *Canarium commune* (Elemi) essential oil (EO) and nanoparticles. Elemi EO is analysed by GC‐FID/MS. The antiproliferative effect of Elemi EO and prepared nanoparticles on human lung adenocarcinoma (A549) and their effect on normal fibroblast cells (CCD‐19Lu) were determined by the MTT test. The levels of TAS, TOS, CYCS, CASP3, TNF‐α and IL‐6 parameters of the experimental groups were determined using specific ELISA. BAX and Bcl‐2 genes were studied with qRT‐PCR to investigate the different ways that cancer cells undergo apoptosis. Limonene (53.7%), a‐phellandrene (14.5%) and elemol (10.1%) were the major components of Elemi EO. 24‐Hour IC_50_ values in the cells were measured for Elemi EO; A549: 1199 μg/mL, CCD‐19Lu: 37.181 μg/mL. TAS and TOS values were found to be higher in cancer cells than in normal cells, and it was found that cancerous cells were dragged into stress and that cancer cells were directed to apoptosis. BAX genes stimulation supported the results. It was determined that Elemi EO and nanoparticles showed anticancer activity without damaging normal cells. Based on these promising results, potential drug candidate Elemi EO loaded nanoparticles may be cell‐specific targeted, oral use possible, new generation nanoparticular drugs.

## 1 INTRODUCTION

When a cell exceeds its growth limits, it turns into a cancer cell. If mutations occur in the genes that control cell proliferation, a cancerous cell is usually formed.^1^ Lung cancer is the leading cause of death in men and women in the world with approximately 2 million diagnoses each year.^2^ The cases about 90% of those occur as a result of smoking, exposure to tobacco use.^3^ Approximately 85% of all lung cancers are classified as non‐small‐cell lung cancer (NSCLC) and of these NSCLCs, around 50% are identified as lung adenocarcinoma based on histology.^4^

It is challenging to create new drugs to stop the growth of human lung cancer cells because of the serious adverse effects, toxicity and high expenses involved.^3^ To address this issue, researchers are examining the impact of medicinal and aromatic plants on lung cancer cells. Many studies have been conducted on the effect of herbal ingredients on human adenocarcinoma cells (A549).^5^, ^6^, ^7^ As herbal ingredients, essential oils have emerged as an alternative solution for various infections, including cancer cells.^3^ They are generally obtained from the fruits, leaves, seeds and bark of aromatic plants.^5^ To date, their antibacterial, antifungal, antiviral,^8^ antioxidant and antitumoral^9^ activities have been examined.

The development of lung cancer treatment methods, supportive treatments and alternative treatment methods are very important in terms of finding a solution to these diseases. For this purpose, this study was conducted to search for a solution that could be effective on lung cancer by investigating essential oil for this problem. Elemi essential oil (EO) was chosen according to the literature review and it is aimed to investigate its anticancer effects. Elemi (*Canarium* L.) is the general name of several oleoresins obtained from different plants in the Burseraceae. The most common of these oleoresins is *Canarium luzonicum* Miq. or *Canarium commune* L. (*Canarium indicum*).^10^ Elemi EO is obtained by distillation of elemi resin. In several studies, the antifungal^11^ and antimicrobial^12^ activities of Elemi were investigated. There are some studies showing its use in arthritis and chest pain. The studies were reporting the use of oleoresin as a poultice in ulcerated wounds.^13^ But in the literature review, no study was found on the anticancer effect of elemi EO. For this reason, the effect of elemi EO and elemi EO loaded nanoparticle formulations on A549 and CCD‐19Lu (human normal lung fibroblast cells) was researched by the MTT test. The levels of TAS and TOS, CYCS, CASP3, TNF‐α and IL‐6 parameters of the experimental groups were determined. Molecular biological analysis by qRT–PCR technique, apoptosis by flow cytometry and wound‐healing effect by scratch assay were performed.

## 2 MATERIALS AND METHODS

### 2.1 Chemicals

Essential Oils: Elemi essential oil from Art de Huile were purchased.

Cell Culture Plate: 96‐well, flat bottom, sterile, capped, disposable plate, TPP, (Cat:92096).

Plate: 8‐well plate suitable for automatic cell counter, Roche Diagnostics (Ref: 05650801001).

Cell Count Dye: trypan blue solution, Biological Industries (03‐102‐1B).

Trypsin/EDTA: trypsin/EDTA solution (0.05%/0.02% w/v), Biochrom AG (Cat: L2143).

Cell Culture Medium: Dulbecco's modified Eagle's medium (DMEM), Sigma‐Aldrich (Cat: D6429).

DMSO: dimethylsulfoxide, sterile, cell culture grade, Appli Chem (A3672).

FBS: foetal calf serum (heat inactivated), Capricorn Scientific (Cat: FBS‐HI‐11A).

Antibiotic: mixture of 10.000 U/mL Penicillin and 10 mg/mL Streptomycin, Sigma‐Aldrich (Cat: P4333‐100 mL).

PBS: phosphate buffer (phosphate‐buffered saline), Sigma (P4417).

MTT: thiazolyl blue tetrazolium bromide 98%, Alfa Aesar (298‐93‐1) Sterile‐disposable, various pipette tips and pipettes, flasks, general cell.

Eudragit RS 100: from Evonik Nutrition and Care GmbH. In the experiments, studies were carried out with water taken from a distilled water device (Sartorius Stedim) and sterilized in an autoclave. All other chemicals and reagents used were of pharmaceutical and analytical grade.

### 2.2 Elemi essential oil analysis

Gas chromatography/mass spectrometer (GC–MS) was used to define the components of Elemi essential oil, and gas chromatography (GC‐FID) was used to determine their relative percentages of the volatiles.

### 2.3 Cell culture experiments

Human lung adenocarcinoma cells (A549, ATCC® CCL‐185TM) and human normal lung cells (CCD‐19Lu, ATCC® CCL‐210TM) from the American Type Culture Collection (ATCC) were used. Sterilisation of all liquids and plastic materials used in the experiments in an autoclave at 121°C, 1.5 atm for 20 min (*Eryiğit A.Ş. Turkey*), sterilisation of glass and metal materials at 180°C for 2 h in a dry air sterilizer (oven, Heraeus). Sterilisation of other liquid solutions was achieved by passing them through sterile disposable injector filters (Sartorius Stedim Biotech) with a pore diameter of 0.22 μm. Studies were conducted in aseptic conditions in a laminar flow cabinet (Mars Scanlaf, Labogene ApS, Denmark), protected from light.

#### 2.3.1 Preparation of cell cultures

Cells pre‐existing in AUBIBAM −196°C liquid nitrogen stocks were placed in appropriate media using standard cell culture techniques (37 ± 1°C containing 5% CO_2_, approximately 95% relative humidity). (90% Medium + 10% Serum + 1% Antibiotic) were first cultured in 25 cm^2^ flasks (BINDER CB 150 E3) in the incubator so that they can grow rapidly. The cells were checked under an inverted type light microscope (Olympus CKX41‐SC30) and passaged in sufficient quantity and ready for analysis in 75 cm^2^ flasks (CCD‐19Lu in the 7th passage, A549) in the 10th passage. Adhere cells on the flask surface were washed two times with phosphate buffer saline (PBS), removed with 1× Trypsin/EDTA solution, neutralized with the medium, transferred to falcon tubes and centrifuged at 1200 *g* for 5 min (HettichLab Teknoloji). Afterward, the supernatant was discarded and 1 mL of medium was added, and it was suspended, ready for counting. After 10 μL of the prepared suspended cell solution was stained with trypan blue at a ratio of 1:1 for 10 s, counting was performed in an automated cell counter (LUNA II; Logos Biosystems). Cells were diluted with medium (1 well of cell‐free medium = blinded) at 10^4^ cells/200 μL of medium per well for each plate. Sterile disposable 96‐well plates with a flat bottom, which can contain up to 250 μL of a liquid solution, were used for analyses.

#### 2.3.2 Preparation of different working concentrations of stock solution

Stock solution: It is the liquid state of which the concentration is determined, prepared by dissolving the required amount of elemi essential oil in a solvent by weighing them on a precision scale (Mettler Toledo).

#### 2.3.3 Preparation of different working concentrations of Elemi essential oil

The essential oil was tested by dissolving them in the cell medium in DMSO solvent and it was observed that it dissolved homogeneously. While preparing the essential oil concentrations to be applied, concentration calculations were made so that the DMSO ratio did not exceed 0.1%. Essential oil concentrations were evaluated by weighing and calculating the essential oil. In order to find the 50% inhibitory concentration (IC_50_) of elemi essential oil in cells, seven different concentrations were applied. Elemi (*Canarium commune)* essential oil 0, 1.5625, 3.125, 6.25, 12.5, 25, 50, 100 μg/mL doses used for 24 h MTT analysis in CCD‐19Lu and A549 cells.

For this, different dilutions on the stock solution were prepared to be carried on the plate at the final concentration (calculated as 1:1 dilution) with the medium suitable for the cell type. All concentrations were obtained by dilutions over the highest concentration and then applied to the plate. Half of the 200 μL medium (100 μL) was drawn from the cells adhered to the bottom of the plate and fresh medium containing different concentrations of elemi essential oil was added to the drawn amount (100 μL).

Analysis evaluation was carried out by calculating *n* = 5 repetitive data, elemi essential oil was kept in suitable conditions until analysis and studied under protection from light. Before mixing the essential oil solutions of different concentrations with the medium, 20 min. It was exposed to direct (90° angle) UV light, and no degradation was observed in essential oil, then mixing with the medium was performed.

### 2.4 Preparation of nanoparticle samples

#### 2.4.1 Preparation of elemi essential oil loaded PLGA‐based nanoparticle formulation

Elemi essential oil loaded PLGA‐based nanoparticles were prepared by the nanoprecipitation method.^14^ To prepare the blank formulation, PLGA (100 mg) and Span 60 (30 mg) were dissolved in 1:1:1 (v:v:v) acetone:ethanol:dimethyl formamide (3 mL). At a rate of 5 mL/h, the prepared solution was dropped into 15 mL of Pluronic F‐68 (0.5%) aqueous solution. Then, the organic solvent was evaporated at room temperature and centrifuged at 11,000 *g* at 4°C for 30 min to collect nanoparticles from the aqueous solution. The collected nanoparticles were dispersed in 15 mL of distilled water. The same process was applied three times to wash the nanoparticles.

For the preparation of PLGA nanoparticles containing Elemi essential oil, 10% (10 mg) of Elemi essential oil was separately weighed and added to the organic phase described in the above paragraph. At a rate of 5 mL/h, the obtained solution was dropped into 15 mL of Pluronic F‐68 (0.5%) aqueous solution. Then, the organic solvent mixture was evaporated at room temperature. To collect nanoparticles from the aqueous solution was centrifuged at 11,000 rpm at 4°C for 30 min After the first centrifugation process was completed, the collected nanoparticles were dispersed in 15 mL distilled water. The same centrifugation process was applied three times to wash the nanoparticles.

#### 2.4.2 Preparation of Elemi essential oil loaded Eudragit RS 100‐based nanoparticle formulation

Elemi essential oil loaded Eudragit RS 100 (ERS)‐based nanoparticles were prepared using the nanoprecipitation method.^14^ To prepare the blank formulation, ERS (100 mg) and Span 60 (30 mg) were dissolved in 3:1 (v:v) ethanol: dimethylsulfoxide (3 mL). This solution obtained was dropped into 15 mL of Pluronic F‐68 (0.5%) aqueous solution at a rate of 5 mL/h. The organic solvent was evaporated at room temperature. To collect nanoparticles from the aqueous solution was centrifuged at a stirring speed of 11.000 rpm. The centrifugation was performed at 4°C for 30 min. After the first centrifugation process was completed, the collected nanoparticles were dispersed in 15 mL distilled water and the same centrifugation process was applied three times to wash the nanoparticles.

For the preparation of ERS nanoparticles containing Elemi essential oil, 10% (10 mg) of Elemi essential oil was weighed separately and added to the organic phase. At a rate of 5 mL/h, this solution was dropped into 15 mL of 0.5% Pluronic F‐68 aqueous solution. Then, the organic solvent was evaporated at room temperature. To collect nanoparticles from the aqueous solution it was centrifuged at a stirring speed of 11,000 rpm. Centrifugation was performed at 4°C for 30 min. The collected nanoparticles were dispersed in 15 mL of distilled water. The same centrifugation process was applied three times to wash the nanoparticles.

#### 2.4.3 Characterisation of nanoparticle formulations

##### Particle size, polydispersity index and zeta potential

Particle size (PS), polydispersity index (PDI) and zeta potential (ZP) of NPs analysed by a Zetasizer Nano ZS (Malvern Instruments).

##### Encapsulation efficiency (EE%)

An indirect method was followed to determine the encapsulation efficiency. The newly prepared nanoparticle dispersion was centrifuged for 30 min at 11,000 rpm, and the supernatants were collected. UV spectroscopy (Shimadzu) was used to determine the amount of free drug. The supernatants were filtered using a 0.45 μm membrane filter, and then they were measured at 264 and 268 nm, respectively.

$$\begin{array}{c} \%\text{Encapsulation efficiency}\\ =\frac{\text{Total oil amount}‐\text{Amount of oil in supernatant}}{\text{Total oil amount}}\times 100. \end{array}$$

### 2.5 MTT cytotoxicity assay

#### 2.5.1 MTT Cytotoxicity assay of Elemi essential oil

After the cells were seeded on the plate, they were allowed to incubate for 24 h and the cells were allowed to adhere to the bottom of the plate. At the end of 24 h, different concentrations of Elemi essential oil solutions were added to the cells. After a 24‐h incubation period, 20 μL of the stock MTT solution (dissolved in 5 mg/mL PBS and filtered MTT solution) was added to each well‐protected from light for 3 hours, and the cells were then completely removed from the medium and 100 μL of DMSO added to the Plate 15. After shaking for 1 min in the dark (orbital shaker, HeidolphUnimax 1010) and after dissolving the formazan crystals, it was read in a multi‐mode plate reader (BioTek Synergy HTX) at a wavelength of 540 nm. Calculations were made and data were obtained.

The data were created by calculating in Microsoft Office Excel. For this, first of all, the blind well value was subtracted from all optical readings. The averages of optical densities, % viability and % inhibition values were made from Excel formula calculations; After finding the ‘cutoff point’ and ‘slope’ values, the results were discussed by taking into account the *r* ^2^ values (automatic calculation was made based on the inhibition and concentration values) in order to find the IC_50_ value. The control group's viability was considered to be 100% and all other viability values were calculated based on that.

#### 2.5.2 MTT cytotoxicity assay of nanoparticle formulations

To examine the cytotoxicity of nanoparticle formulations of elemi‐loaded essential oil and not containing any oil (placebo), the same concentrations were applied to both cell types. The necessary calculations were made by weighing the nanoparticles with known essential oil content according to the loading capacity. Seven different concentrations were prepared by diluting the formulations that were weighed to contain a maximum of 10 mg/mL essential oil. The nanoparticle formulation containing Elemi (*Canarium commune*) essential oils 0, 1.5625, 0.3125, 0.625, 1.25, 2.5, 5, 10 μg/mL doses used for 24 h MTT analysis in CCD‐19Lu and A549 cells.

### 2.6 Preparation of cell lysates

Cell lysates were prepared for the purpose of determining the total antioxidant status (TAS), total oxidant status (TOS), caspase 3 (CASP3) and cytochrome C somatic (CYCS), as well as for conducting qRT‐PCR tests. Adhere on the flask surface cells were washed two times with PBS, they were removed with 1× Trypsin/EDTA solution, neutralized with the medium and transferred to falcon tubes and centrifuged for 5 min at 1200 rpm (HettichLab Technology). After washing the cells in cold PBS three times, they were suspended and stored for 2 h at 4°C in a new lysis buffer containing 20 mM EDTA, 1 mg/mL proteinase K, 10 mM Tris–HCl at pH 8.0, 1 mM dithiothreitol and 50 mM HEPES at pH 7.0. To eliminate the cellular debris, the suspended cells were then centrifuged for 10 min at 16,000 g and 4°C. Prepared cell lysates were used immediately for experiments and analysis.

### 2.7 Biochemical experiments

The levels of TAS and TOS, CYCS, CASP3, TNF‐α and IL‐6 parameters of the experimental groups were determined by ELISA kits. Studies were performed using an ELISA reader (BIOTEK ELx808) and an ELISA plate washer (BIOTEK ELx50). Results are reported as mean ± standard deviation, each performed three times.

#### 2.7.1 Oxidative stress marker experiments

##### Measuring total antioxidant status

The total antioxidant levels of the cell samples were determined using the total antioxidant status (tas) kit (Catalogue no. RL0017) produced by Rel Assay in Gaziantep, Turkey. The kit operates on the principle of reducing the coloured 3‐ethylbenzothiazoline‐6‐sulfonate (ABTS) to its colourless form through the action of antioxidants. In the working procedure of the test, plates not coated with antibody were used. 18 μL of each of the standard, blank and samples were added to the relevant wells on the plates. After adding 300 μL of Reagent 1 to each well, the first absorbance value of the resulting mixture was determined at 660 nm. Then, 45 μL of Reagent 2 was added to each well and incubated for 5 min at 37°C. After incubation, the final absorbance value was read at 660 nm and recorded. Results are reported as mmol Trolox Equiv/L.

##### Measuring total oxidant status

Total oxidant levels of the samples were determined using the Rel Assay brand total oxidant status (TOS) kit (Catalogue no; RL0024, Rel Assay). The test principle is based on the oxidation process in the cells. In the working procedure of the test, plates not coated with antibody were used. After adding 300 μL of Reagent 1 to each well, the initial absorbance value of the resulting mixture was determined at 530 nm. Then, 15 μL of Reagent 2 was added to each well and incubated at 37°C for 5 min. After incubation, the final absorbance value was read at 530 nm and recorded. The results were reported as μmol H_2_O_2_ Equiv/L.

##### Determination of OSI values

The oxidative stress index (OSI) was calculated as the ratio of TOS to TAS. The TAS value was converted to mmol/L, and the OSI was determined using the following formula: OSI = TOS (μmol H_2_O_2_ Eq/L)/TAS (mmol Trolox Eq/L).

#### 2.7.2 Apoptotic biomarkers

##### Cytochrome C somatic and Caspase‐3 assays

To evaluate the apoptotic markers, the activities of CASP3 and CYCS were measured in the cell samples using relevant kits provided by Cloud‐Clone Corp. with the catalogue numbers SEA594Ra and SEA626Ra, respectively. The concentrations of CASP3 and CYCS were reported in ng/mL.

#### 2.7.3 Proinflomatuary factors assay

The levels of IL‐6 and TNF‐α were measured using a relevant kit supplied by Cloud‐Clone Corp. with the catalogue numbers SEA133Ra and SEA079Ra, respectively. The concentrations of IL‐6 and TNF‐α in the cell lysates were expressed in pg/mL and were determined by comparing with the optical density of the standard curve.

### 2.8 Advanced molecular biological analysis

#### 2.8.1 qRT–PCR technique

The RNA isolation protocol from the sample cells was performed using the EZ‐10 DNAaway kit. The quality and quantity of the isolated RNA samples were assessed with the Thermoscientific NanoDrop instrument (RNA Mini‐Preps kit, BS88133; Bio Basic). cDNA synthesis was carried out according to the manufacturer's instructions using the OneScript® Plus cDNA Synthesis kit (Applied Biological Materials Inc., Cat. No: G234). Real‐time PCR analysis was done using the Bright Green 2× qPCR MasterMix kit (Applied Biological Materials Inc., Cat. No: Mastermix‐U). The results were analysed with the Step One Plus RT‐PCR device and software program. The mRNA expression levels of the target genes were determined from the individual amplification results (*C*_t_ values) and the relative quantification was calculated using the formula $2^{-\Delta \Delta C_{T}}$, with B‐actin as an endogenous control for normalisation. Primer sequence was as follows from forward to reverse; B‐actin; 5′‐GGGCAACATAGCACAGCTTCT‐3′, 5′‐GCTTCACCACCACAGCTGAGA‐3′, Bcl‐2; 5′‐ATGTGTGTGGAGAGCGTCAACC‐3′, 5′‐TGAGCAGAGTCTTCAGAGACAGCC‐3′, BAX; 5′‐CGGGTTGTCGCCCTTTTCTA‐3′, 5′‐TGGTTCTGATCAGTTCCGGC‐3′.

#### 2.8.2 Apoptosis determination studies by flow cytometry

In this test, cells without any fluorescent labelling (FITC− /PI−) were in the Q1‐LL quadrant; FITC‐stained but not PI‐stained, that is, apoptotic or early apoptotic cells (FITC + /PI−) with Q1‐LR quadrant; that is, late apoptotic or necrotic cells (FITC + /PI+) stained with both dyes with the Q1‐UR quadrant; necrotic and dead cells (FITC− /PI+) that were not stained with FITC but stained with PI were shown with the Q1‐UL quadrant. The effects of IC50 values on cells were measured using CytExpert software (Version: 2.2.0.97) on a Beckman Coulter CytoFLEX model flow cytometry device.

Apoptosis determination on cells of IC50 concentrations found by the MTT method was performed according to the procedure in BD Pharmingen's apoptosis kit (BD Annexin V‐FITC Apoptosis Detection Kit I).

Cells at the appropriate density were removed from the flasks and counted (as in the MTT method). After counting, they were seeded in flat‐bottomed, 6‐well plates at 1 × 10^6^ cells per plate and incubated overnight for cells to adhere to the plate bottom. The next day, the substances (CC NP, CC) were treated with IC_50_ concentrations (Placebo formulation was applied as much as the maximum given charged nanoparticle). After the appropriate incubation period (24 h), the cells were removed and centrifuged as in passage. With cold PBS washed cells twice and were transferred to 5 mL tubes to be used in flow cytometry as 1 × 10^5^ cells in 100 μL of 1× binding buffer, diluted homogeneously and suspended. 5 μL PI dyes and 5 μL Annexin V‐FITC were added to tubes, mixed gently and then kept in the dark for 15 min at room temperature, protected from light. At the end of this process, 400 μL of 1× binding buffer was put into each tube and read in the device within 1 h.

### 2.9 Wound healing effect test (Scratch assay)

To observe the effects of the cell population on the wound‐healing process and to examine the possible changes in the migration properties of the cells, CCD‐19Lu cells were seeded in 24‐well culture plates at 100,000 cells/well (by removing and counting as in MTT). When the cells completely covered the bottom of the plate, a slit model was created in the middle of the container under sterile conditions with the help of a 200 μL pipette tip. The closure rate and spacing of this created slit were measured at 12, 24 and 48 h with the help of a light microscope.

### 2.10 Statistical analysis

All cell experiments were performed in three different replicates. Data were evaluated using Kolmogorov–Smirnov and Shapiro–Wilk normality tests. For normally distributed data, the difference between the experimental groups was demonstrated using a one‐way anova test. The differences between the experimental groups, which emerged as a numerical value (p) as a result of all statistical applications, were considered significant at *p* < 0.05 significance level. Tukey test was used for multiple comparisons. Statistical analysis was performed using SPSS Version 21.0 and Graphpad 7 Prism programs. Normally distributed data obtained from all groups were given as mean ± standard error.

## 3 RESULTS

### 3.1 GC–MS results

GC‐FID/MS results of Elemi essential oil are shown in Table 1.

**TABLE 1:** Elemi essential oil composition.

No	Compound^a^	Relative percentage (%)
1	Sabinene	4.0
2	α‐Phellandrene	14.5
3	Limonene	53.7
4	β‐Phellandrene	2.1
5	*p*‐Cymene	2.0
6	Terpinolene	1.0
7	α‐Terpineol	2.7
8	Elemol	10.1
9	Elemicin	3.5
	Total	93.6

^a^ ≥0.5%.

### 3.2 Nanoparticle results

The data obtained regarding the particle size, distribution and zeta potential of the obtained nanoparticles are shown in Tables 2, 3, 4.

**TABLE 2:** Encapsulated essential oil amounts of Elemi essential oil loaded PLGA and Eudragit RS 100‐based nanoparticles.

	PLGA nanoparticle	Eudragit RS 100 nanoparticle
Elemi essential oil	%20.194 ± 1.395	%30.706 ± 2.196

**TABLE 3:** Particle size, distribution and zeta potential PLGA of the obtained nanoparticles.

Formulation	Particle size (nm)	Polydispersity index	Zeta potential (mV)
Empty nanoparticle formulation (placebo)	144.667 ± 0.503	0.186 ± 0.016	−24.400 ± 1.646
Nanoparticle formulation containing elemi essential oil	188.900 ± 3.568	0.259 ± 0.002	−23.300 ± 1.992

**TABLE 4:** Particle size, PDI and zeta potential consequences of Eudragit RS 100 nanoparticles.

Formulation	Particle size (nm)	Polydispersity index	Zeta potential (mV)
Empty nanoparticle formulation (placebo)	265.833 ± 4.196	0.239 ± 0.029	+23.200 ± 1.473
Nanoparticle formulation containing Elemi essential oil	265.467 ± 6.671	0.258 ± 0.052	+23.533 ± 0.709

### 3.3 MTT results

For A549 and CCD‐19Lu cells, the IC_50_ values obtained from the 24 h MTT analysis results of Elemi essential oil and nanoparticle formulation containing elemi essential oil are shown in Tables 5 and 6.

**TABLE 5:** IC_50_ values (μg/mL) for A549 and CCD‐19Lu cells obtained from 24 h MTT analysis results applied to Elemi essential oil.

	CCD‐19Lu		A549	
Test substance	IC_50_ value	*r* ^2^ Value	IC_50_ value	*r* ^2^ Value
Elemi (*Canarium commune*) essential oil	37.181	0.937	1.199	0.942

**TABLE 6:** IC_50_ values (μg/mL) of Elemi essential oil loaded nanoparticle on cells after 24 h of incubation.

	CCD‐19Lu		A549	
Test substance	IC_50_ value	*r* ^2^ Value	IC_50_ value	*r* ^2^ Value
Elemi (*Canarium commune*) essential oil‐loaded NP	6.106	0.971	0.141	0.906

**FIGURE 1:**
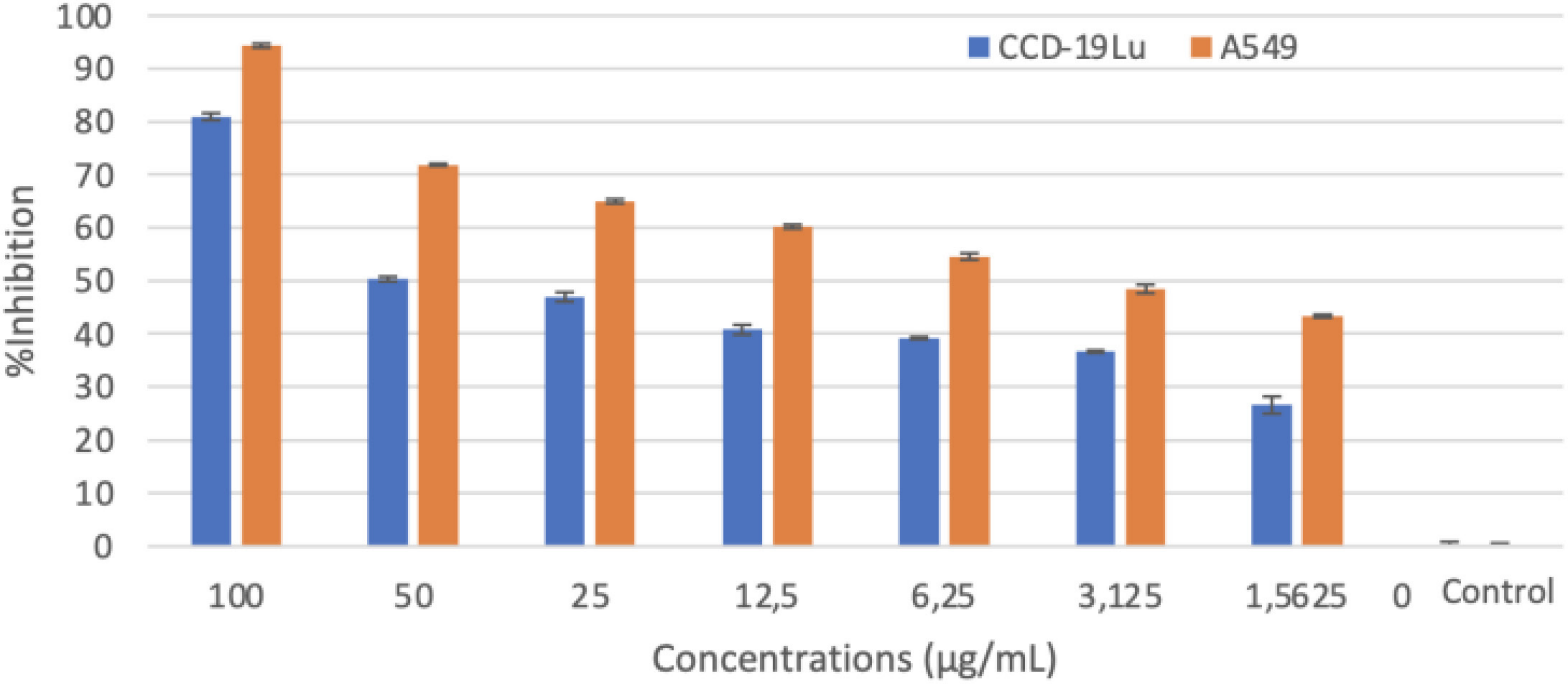
24 h MTT analysis result and % inhibition values for CCD‐19Lu and A549 cells of Elemi (*Canarium commune*) essential oil.

**FIGURE 2:**
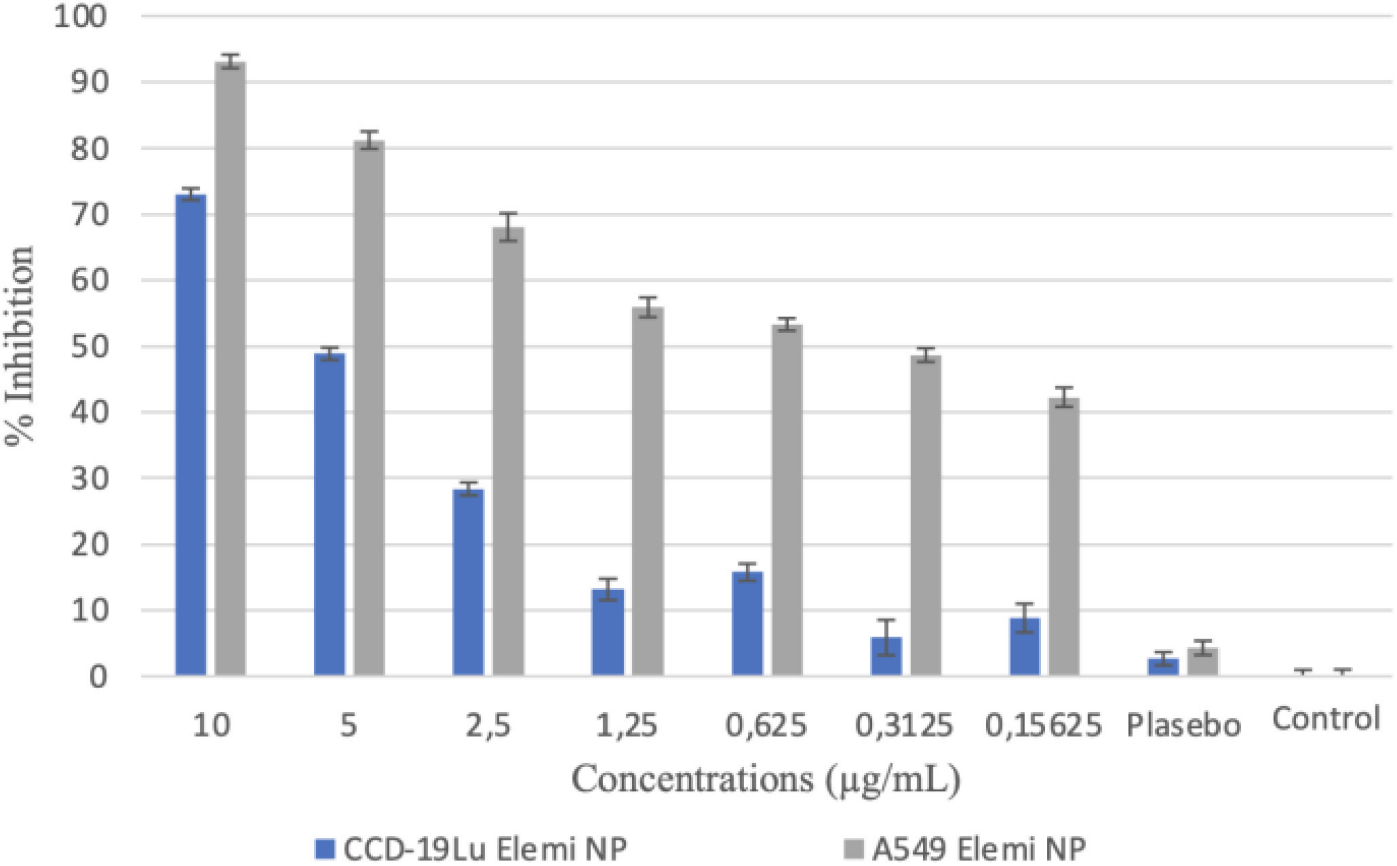
24 h MTT analysis result for CCD‐19Lu and A549 cells of nanoparticle formulation containing Elemi (*Canarium commune*) essential oils.

The graph of % inhibition from the MTT analysis results after 24 h of incubation with Elemi essential oil and nanoparticle formulations containing essential oils on A549 and CCD‐19Lu cells is shown in Figures 1 and 2.

### 3.4 Morphological results

**FIGURE 3:**
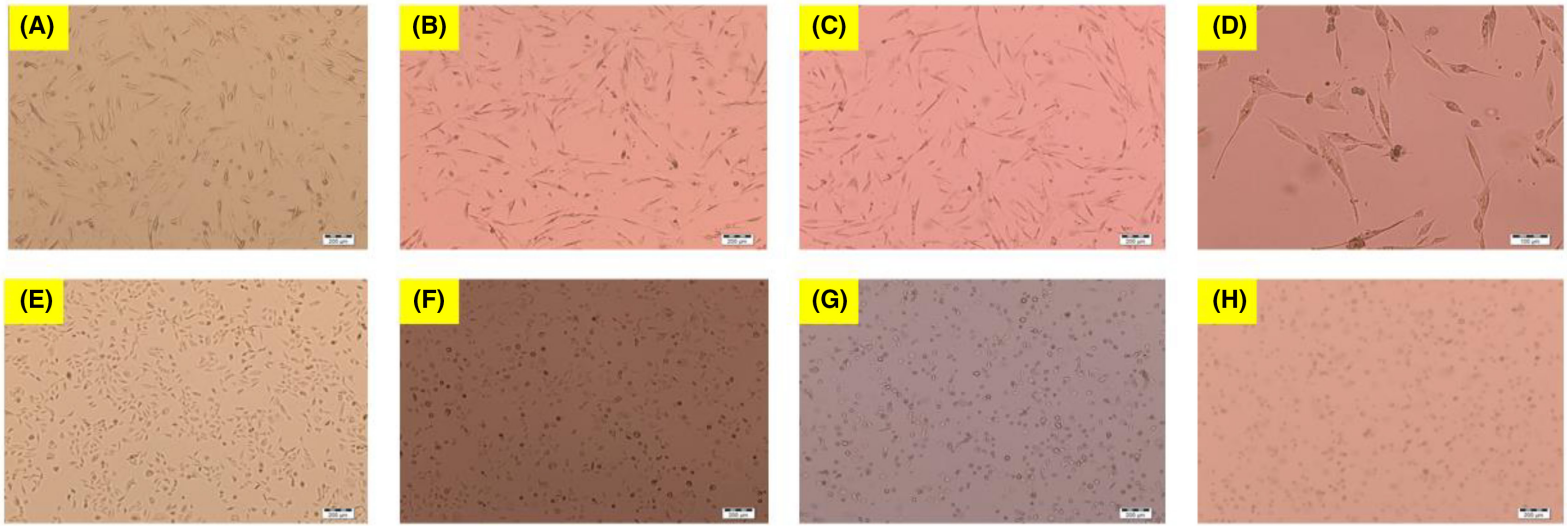
Inverted microscope images of the A549 and CCD‐19Lu cells. A–E: Control, B–F: IC_25_ Elemi, C–G: IC_50_ Elemi. D–H: IC_75_ Elemi‐treated groups. It was observed that the concentration of cells decreased and the number of round and shrunken cells increased in a manner proportional to the concentration in cells treated with elemi. Objective magnification is ×4.

The morphological results obtained by examining the cells with an inverted type light microscope (Olympus CKX41‐SC30) are as in Figure 3. Microscope images are of samples at 4× magnification. A–D figures (Control, IC_25_, IC_50_ and IC_75_) in CCD‐19Lu, E–H Figures (Control, IC_25_, IC_50_ and IC_75_) in A549 cells.

### 3.5 Biochemical results

**FIGURE 4:**
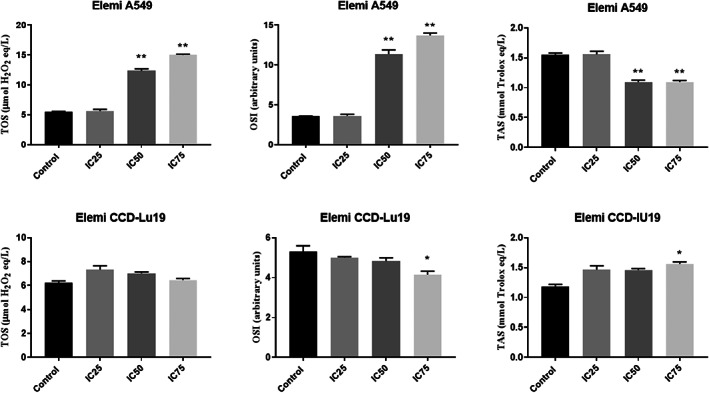
Oxidant molecule and antioxidant levels in A549 and CCD‐19Lu cells treated with Elemi. **p < 0.01 compared with the control *p < 0.05 compared with the group between control. The data is the average of three sets of 96‐well plate measurements, with a standard deviation shown (*n* = 3).

The levels of total antioxidant (TAS) and total oxidant (TOS) for cell lysates treated with IC_25_, IC_50_, and IC_75_, as determined by the MTT analysis method, are displayed in Figure 4 for both cell types. The treatment of A549 cells with elemi at IC_50_ and IC_75_ doses led to a significant rise in their total oxidant (TOS) levels. In comparison, the treatment of elemi at the IC_25_ dose caused a slight increase in TOS levels, which was not statistically significant compared to the control. Moreover, TOS levels did not show a dose‐dependent change in CCD‐19Lu cells.

**FIGURE 5:**
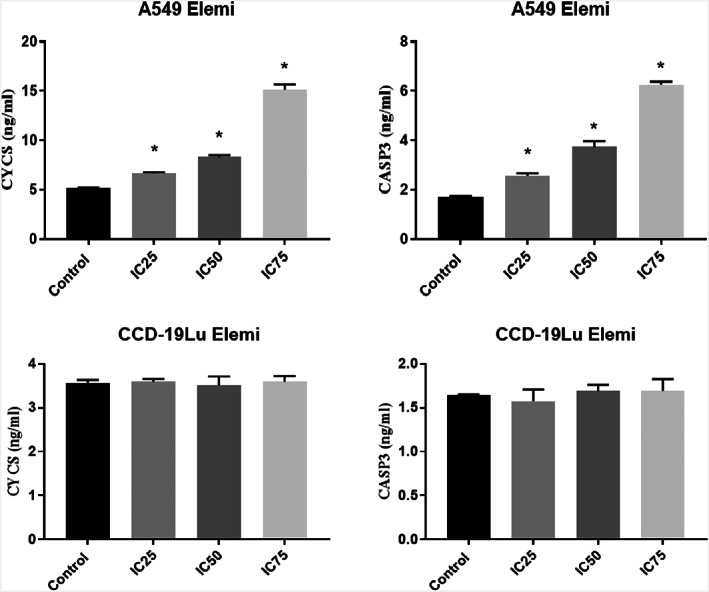
Caspase 3 (CASP3) and cytochrome C somatic (CYCS) levels activities in A549 cancer cell and CCD‐19Lu treated with Elemi essential oil *p < 0.05 compared with the group between control. The data are the average of three sets of 96‐well plate measurements, with a standard deviation shown (*n* = 3).

We discovered that the treatment with Elemi led to a significant rise in the CYCS level and the activity of CASP3, thereby triggering apoptotic pathways in A549 cells as shown in Figure 5.

**FIGURE 6:**
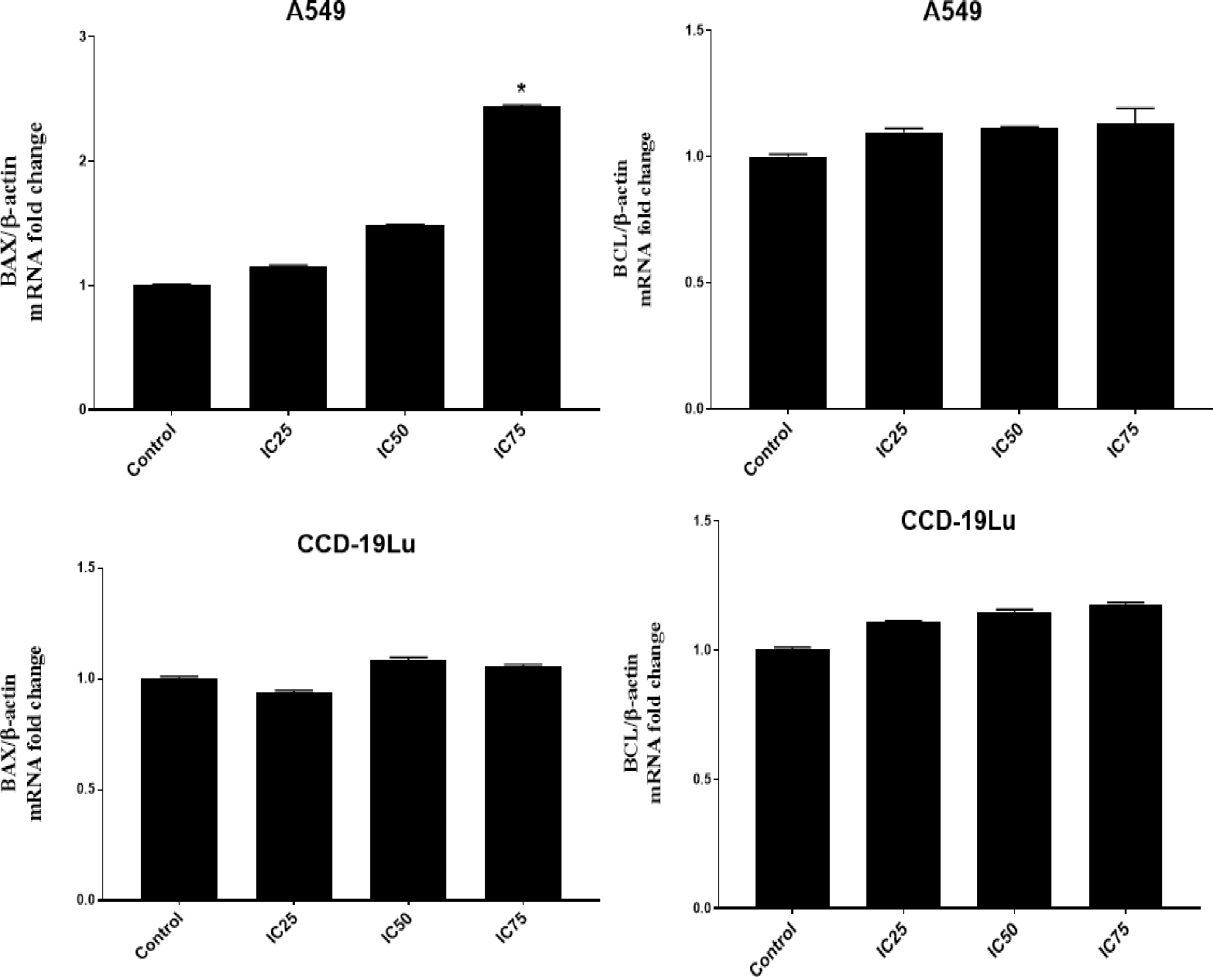
BAX and BCL‐2 mRNA expression levels for A549 and CCD‐19Lu cells treated with Elemi dose‐dependently. *p < 0.05 compared with the group between control. The data is the average of three sets of 96‐well plate measurements, with a standard deviation shown (*n* = 3).

BCL‐2 (anti‐apoptotic protein) and BAX (apoptotic protein) mRNA expression in A549 and CCD‐19Lu cells are shown in Figure 6. As for BAX, A549 cells with IC_75_ Elemi EO increased about twofold change. However, BCL‐2 mRNA expression is unchanged.

**FIGURE 7:**
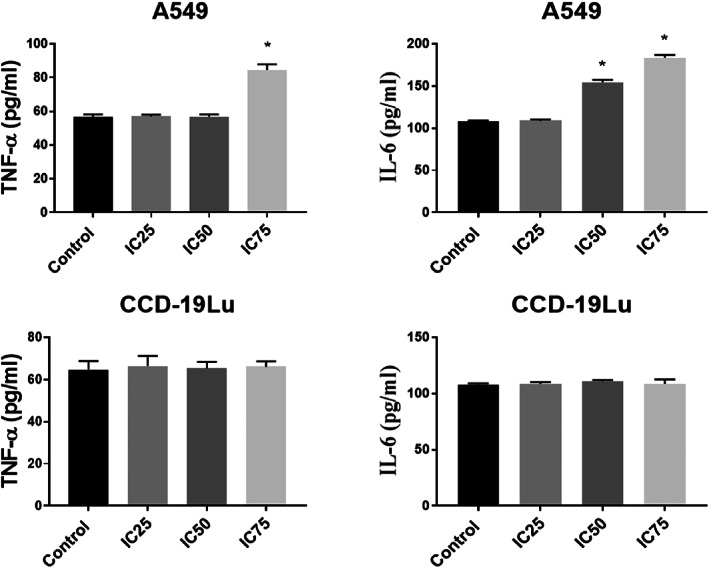
Interleukin‐6 (IL‐6) levels and tumour necrosis factor‐alpha (TNF‐α) in A549 lung cancer cells and CCD19‐Lu treated with different elemi concentrations. *p < 0.05. The data are the average of three sets of 96‐well plate measurements, with a standard deviation shown (*n* = 3).

The effects of different concentrations of elemi on the levels of TNF‐α and IL‐6 in A549 and CCD19‐Lu cells are presented in Figure 7. The IC75 Elemi treatment led to a significant increase in the TNF‐α and IL‐6 levels compared to the control (*p* < 0.05). However, the IC25 Elemi treatment did not result in a significant change in TNF‐α and IL‐6 levels in A549 cells. Proinflammatory cytokine levels did not change in CCD19‐Lu cells.

### 3.6 Flow cytometry results

**FIGURE 8:**
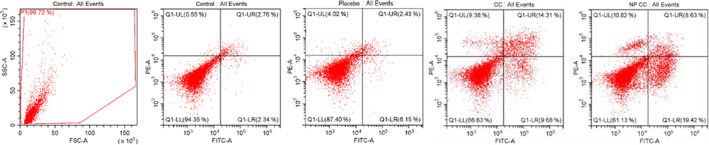
Annexin V‐FITC/PI of 24 h IC_50_ values in A549 cells.

To observe that the device readings are correct and to show that there is no scattering, the negative control group (with cells, without dye) and cell‐free media of the substances were analysed in the device and no effects or deviations that could change the test results were observed. The rates of early apoptosis, late apoptosis and necrosis on cells of the IC_50_ values found were evaluated. According to the results obtained from apoptotic cell determination, Elemi was found to be approximately apoptotic (Figure 8; Table 7).

**TABLE 7:** Flow cytometry analysis results for apoptosis detection of NPs on A549 cells (%) (CC: *Canarium commune*; NP CC: Nanoparticule *Canarium commune*).

Test substance	Necrotic or dead Cells (UL)	Late apoptotic cells (UR)	Unstained cells (LL)	Early apoptotic cells (LR)	(UR + LR)
CC	9.38	14.31	66.63	9.68	23.99
NP CC	10.82	8.63	61.13	19.42	28.05
Placebo	4.02	2.43	87.40	6.15	8.58
Control	0.55	2.76	94.35	2.34	5.1

### 3.7 Wound healing effect test (Scratch assay) results

**FIGURE 9:**
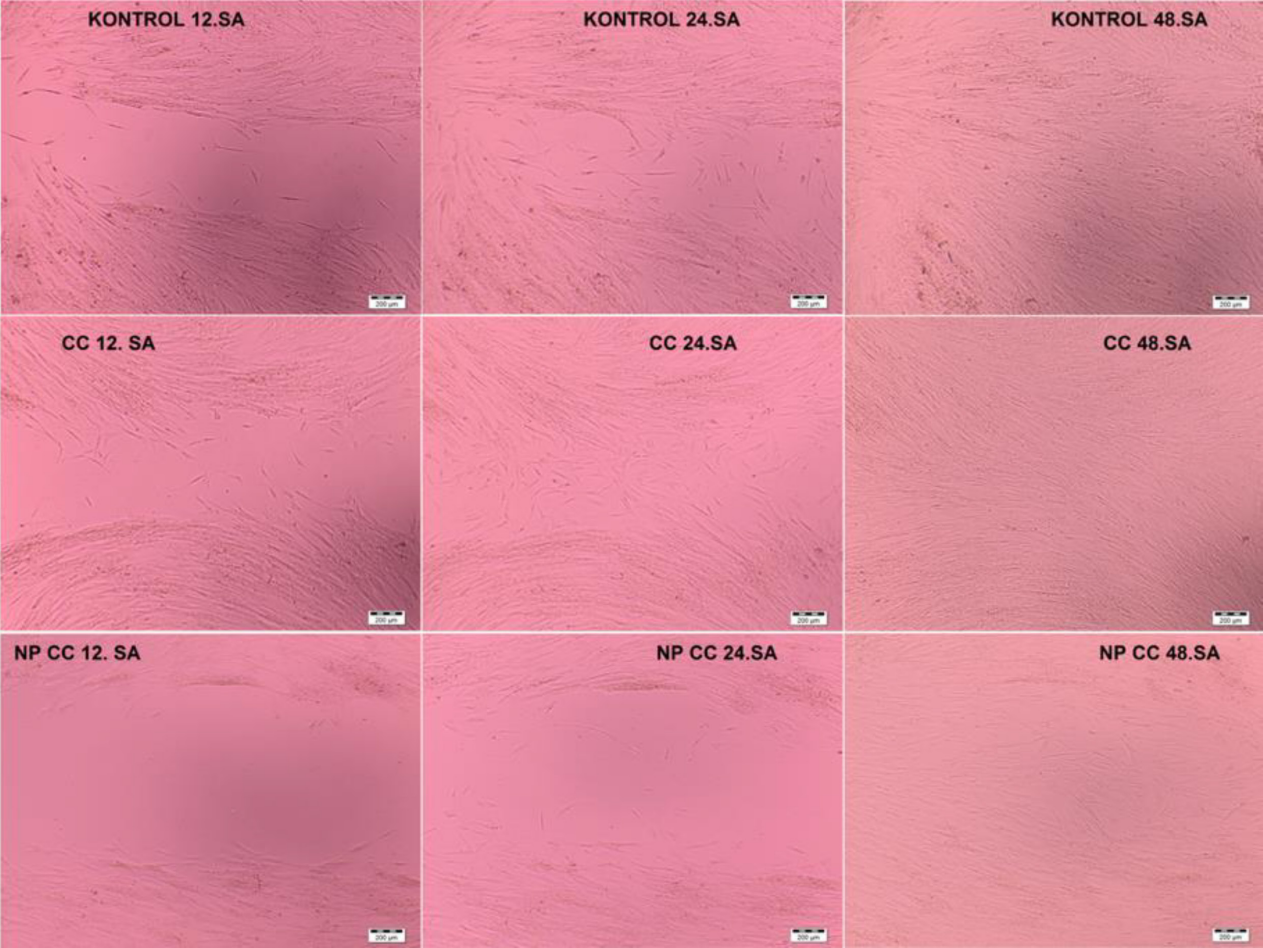
Light microscope images of CCD‐19Lu cells wound‐healing by the Elemi (Scratch assay) (Scale magnification: 200 μm, 10× optical magnification).

Although the migration tendency of NPs was different from the elemi essential oil, it was observed that complete closure was as fast as the control group cells. It was determined that elemi had a higher wound‐healing potential than the control group (Figure 9).

## 4 DISCUSSION

Major components of Elemi essential oil are limonene (53.7%), α‐phellandrene (14.5%), elemol (10.1%), sabinene (4%), elemicin (3.5%), α‐terpineol (2.7%), β‐phellandrene (2.1%), *ρ*‐cymene (2%), terpinolene (1%) (Table 1). GC—FID/MS analysis results are consistent with the literature review content finding.^15^

The particle size, PDI and zeta potential results of the developed formulations are given in Tables 6 and 7. While the average particle size of the empty PLGA nanoparticle was 144.667 nm, the average particle size of elemi essential oil loaded PLGA nanoparticles was 188.900 nm, respectively (Table 6). When the empty PLGA was examined, it was reported that the particle size increased when the essential oil was loaded into the nanoparticles. Considering previous studies,^14^, ^16^ it is known that the particle sizes of nanoparticles increase as a result of drug active ingredient/essential oil loading. The average PDI of the empty nanoparticle is 0.186, whereas the average PDI of Elemi EO‐loaded PLGA nanoparticles is 0.259, respectively (Table 6). PDI, a ratio that gives information about the homogeneity of the nanoparticle system, reflects the quality of the nanoparticle distribution in the range of 0.0–1.0. PDI values of <0.1 indicate the highest dispersion quality. Most authors consider PDI values < 0.3 to be optimum; however, values < 0.5 are also acceptable.^17^ According to the literature, it can be said that monodisperse and high‐quality nanoparticles were produced in this study. A colloidal system with a zeta potential value of ±30 mV is considered a stable formulation when dispersed as a colloidal dispersion in a liquid. Zeta potentials between −5.0 and −15.0 mV are in the region of limited flocculation; and the maximum zone of flocculation between −5.0 and −3.0 mV has been reported previously.^18^ Particle size, PDI and zeta potential results of nanoparticles prepared with Eudragit RS 100 (ERS) are given in Table 7. Nanoparticles prepared with ERS are in nanosize like those prepared with PLGA, and it is noticeable in the table that they are monodisperse particles. The difference is that nanoparticles prepared with ERS should have positive zeta potential. This is due to the polymer. Positive zeta potential is a very important parameter in terms of interaction with cells.^19^

Elemi essential oil IC_50_ values are 1.199 μg/mL on A549, and 37.181 μg/mL on CCD‐19Lu (Table 2). The % inhibition values of CCD‐19Lu cells and A549 cells are given in Figures 1 and 2. Morphological results of elemi essential oil A549 and CCD‐19Lu in a light microscope (10×) at IC_50_ values are seen in Figure 3. In these results, when the normal cells are compared with the control group, there is no significant difference between the application images of elemi essential oil (IC_50_ values applied on A549). The shapes of the cells are the same as in the control group, no morphological changes are observed. When compared with the control group, Elemi essential oil caused apoptosis in A549 cells morphologically. MTT and morphological results support that Elemi essential oil shows their antiproliferative activities without damaging healthy cells at IC_50_ values. There are some studies in the literature with some species belonging to the genus *Canarium* L., but no anticancer studies have been found. Studies on species belonging to the genus *Canarium* L. have been reported in Mogana and Wiart.^10^ Dongmo et al.^20^ in their study with the lipoxygenase method found the IC_50_ for *Canarium schweinfurthii* as 62.6 ppm. Koudou et al.^21^ found analgesic effective at doses 1, 2 and 3 mg/kg i.p. In the literature review, IC_50_ values of some essential oils on A549 were reached; 90.0 ± 5.0 μg/mL for *Vitex agnus castus* L. berries essential oil,^22^ 79.83 ± 0.856 μg/mL for *Pistacia lentiscus* essential oil,^23^ 1.73 ± 0.37 μg/mL for *Cymbopogon citratus* Stapf essential oil.^7^ When the IC_50_ values found in the literature are compared with elemi essential oil values in this study, it can be interpreted that it is more effective on A549.

The results of the biochemical analysis are shown in Figures 4, 5, 6, 7. It is seen that CASP3 and CYCS values at IC_25_, IC_50_ and IC_75_ concentrations are statistically significant compared to the control group (*p* < 0.05). Elemi increased the CASP3 value approximately 2.1 times in IC_50_; approximately 3.6 times the CASP3 value in IC_75_; approximately 1.6 times the CYCS value in IC_50_; approximately 2.9 times the CYCS value in IC_75_ in A549 cells compared to the control group. As a result, it can be said that Elemi essential oil leads A549 cells to apoptosis by increasing IC_50_ values and increasing concentrations of apoptotic proteins CASP3 and CYCS. In Figure 4, it is seen that A549 elemi OSI values are statistically significant at IC_50_ and IC_75_ concentrations compared to the control group (p < 0.01). On the other hand, elemi CCD‐19Lu OSI values do not show a statistically significant difference in IC_50_ concentration compared to the control group. Elemi increased the OSI value approximately 3.2 times in IC_50_ and approximately 3.9 times in IC_75_ compared to the control group in elemi A549 cells. According to these results, it can be said that elemi essential oil leads A549 cells to apoptosis by increasing oxidative stress on A549 at IC_50_ and IC_75_ values. The qRT‐PCR technique results are shown in Figure 6. According to these results, when compared to the control group, the Bax fold change values of elemi essential oil at IC_75_ concentration in A549 cells were statistically significant (*p* < 0.05). There is no significant difference in Bcl‐2‐fold change values IC_25_, IC_50_ and IC_75_ concentrations compared to A549 elemi and control group. Compared to the CCD‐19Lu elemi and the cypress control group, there is no significant difference in Bax and Bcl‐2‐fold change values, IC_25_, IC_50_ and IC_75_ concentrations. According to these results, it can be said that Elemi essential oil stimulates apoptosis by activating Bax genes. Similarly, The disruption of mitochondrial membrane potential by essential oil causes an elevation in ROS levels and a decline in GSH levels, which in turn leads to various disturbances in the Bcl/Bax ratio and an augmentation of caspase 3 and caspase 9 activity. The antitumor properties of a camphene derived from the essential oil of the *Piper cernuum* plant were examined in melanoma cells. The research revealed that this compound was capable of triggering apoptosis through the activation of the caspase‐3 pathway.^24^ Through the modulation of mitochondrial membrane permeability, carvacrol effectively triggered apoptosis in the MDA‐MB‐231 cell line, a metastatic breast cancer model. This process led to the activation of caspases, as evidenced by the release of cytochrome C, cleavage of poly ADP ribose polymerase (PARP), and fragmentation of DNA.^25^ The study aimed to examine the cytotoxic effects of Elemi oil (EO) containing volatile oil on L929 cells. Similar activity was observed in HT‐29 epithelial cells when exposed to escalating concentrations of EO‐containing essential oils (25–200 g/mL), indicating that EO exhibited strong cytotoxic effects on the mitochondrial activity of the cells.^26^ Yu et al. found that the expression of genes related to apoptosis and autophagy increased in tumours after treatment with d‐limonene. Yu et al. support the work of Zhang et al. investigated the synergistic effects of Paclitaxel, which is used in the treatment of small‐cell lung cancer, with the combination of α‐pinene and β‐pinene and reached a more effective result and apoptosis results in their studies.^27^, ^28^ Elemi demonstrated a significant reduction in the proliferation of PC3 and LNCaP prostate cancer cells.^29^ No study could be found in the literature that showed the anticancer potential of Elemi essential oil and nanocapsulation formulation on A549 and CCD‐19Lu cells.

## 5 CONCLUSION

When all the results are examined, it is seen that the zeta potential values are not at the aggregation limit. Accordingly, it can be said that the stability of all PLGA nanoparticles prepared in this study is good. It is thought that the zeta potentials of the nanoparticles found may be cell‐specific targeted, oral use possible, and new generation nanoparticular drugs.

It was considered as a potential new generation drug candidate at IC_50_ concentrations with elemi essential oil. To increase the effectiveness of elemi essential oil, to make them more effective when using them at a lower rate, and to facilitate the application, the nanoparticular formulation was preferred and elemi nanoparticular formulation was developed.

According to the results obtained from cytotoxicity analyses of NPs, essential oil activity increased with NP formulation (*p* < 0.05). On the other hand, the placebo formulation did not have a negative effect on cell proliferation. In conclusion, analyzes of apoptotic and oxidative markers show that apoptotic pathways are stimulated. However, decreased oxidative factors were measured in CCD‐19Lu cells. The results show the usability of Elemi essential oil for anticancer activity.

## AUTHOR CONTRIBUTIONS

**Beril Kurban:** Conceptualization (equal); data curation (equal); funding acquisition (lead); investigation (equal); methodology (equal); project administration (equal). **Tuğba Tuncel:** Conceptualization (equal); data curation (equal); funding acquisition (equal); investigation (equal); methodology (equal); project administration (equal); resources (equal); supervision (equal); writing – original draft (equal); writing – review and editing (equal). **Şennur Görgülü:** Investigation (equal). **Fatih Kar:** Conceptualization (equal); investigation (equal); methodology (equal); writing – original draft (equal); writing – review and editing (equal). **Alper Öztürk:** Investigation (equal). **Temel Özek:** Supervision (equal).

## CONFLICT OF INTEREST STATEMENT

The authors declare that there are no conflicts of interest.

## Data Availability

Data will be made available on request.

## References

[1] Majumder D , Debnath R , Maiti D . IL‐27 along with IL‐28B attenuates the pulmonary redox impairment, inflammation and immunosuppression in benzo(a)pyrene induced lung cancer bearing mice. Life Sci. 2020;260:118384. doi:10.1016/j.lfs.2020.118384 32898529

[2] Wang J , Li X , Chen H . Organoid models in lung regeneration and cancer. Cancer Lett. 2020;475:129‐135. doi:10.1016/j.canlet.2020.01.030 32032677

[3] Rajivgandhi G , Saravanan K , Ramachandran G , et al. Enhanced anti‐cancer activity of chitosan loaded *Morinda citrifolia* essential oil against A549 human lung cancer cells. Int J Biol Macromol. 2020;164:4010‐4021. doi:10.1016/j.ijbiomac.2020.08.169 32853609

[4] He M , Li K , Yu C , et al. In vitro study of FUZ as a novel potential therapeutic target in non‐small‐cell lung cancer. Life Sci. 2018;197:91‐100. doi:10.1016/j.lfs.2018.02.007 29421438

[5] Niu QL , Sun H , Liu C , et al. *Croton tiglium* essential oil compounds have anti‐proliferative and pro‐apoptotic effects in A549 lung cancer cell lines. PLoS One. 2020;15(5):e0231437. doi:10.1371/journal.pone.0231437 32357169 PMC7194401

[6] Rad EY , Tabrizi MH , Ardalan P , et al. Citrus lemon essential oil nanoemulsion (CLEO‐NE), a safe cell‐depended apoptosis inducer in human A549 lung cancer cells with anti‐angiogenic activity. J Microencapsul. 2020;37(5):394‐402. doi:10.1080/02652048.2020.1767223 32400238

[7] Trang DT , Hoang TKV , Nguyen TTM , et al. Essential oils of lemongrass (*Cymbopogon citratus* Stapf) induces apoptosis and cell cycle arrest in a549 lung cancer cells. Biomed Res Int. 2020;2020:5924856. doi:10.1155/2020/5924856 32420353 PMC7201560

[8] Tariq S , Wani S , Rasool W , et al. A comprehensive review of the antibacterial, antifungal and antiviral potential of essential oils and their chemical constituents against drug‐resistant microbial pathogens. Microb Pathog. 2019;134:103580. doi:10.1016/j.micpath.2019.103580 31195112

[9] Fitsiou E , Pappa A . Anticancer activity of essential oils and other extracts from aromatic plants grown in Greece. Antioxidants. 2019;8(8):290. doi:10.3390/antiox8080290 31394842 PMC6720353

[10] Mogana R , Wiart C . *Canarium* L.: a phytochemical and pharmacological review. J Pharm Res. 2011;4(8):2482‐2489.

[11] Angelini P , Bricchi E , Zeppilli N , et al. Screening of the antifungal activity of essential oils against human and plant pathogenic filamentous fungi. Fl Medit. 2019;29:5‐12. doi:10.7320/FlMedit29.005

[12] Kačániová M , Terentjeva M , Štefániková J , et al. Chemical composition and antimicrobial activity of selected essential oils against Staphylococcus spp. isolated from human semen. Antibiotics. 2020;9(11):765. doi:10.3390/antibiotics9110765 33142792 PMC7693587

[13] Murthy KSR , Reddy MC , Rani SS , Pullaiah T . Bioactive principles and biological properties of essential oils of Burseraceae: a review. J Pharmacognosy Phytochem. 2016;5:247.

[14] Öztürk AA , Yenilmez E , Özarda MG . Clarithromycin‐loaded poly (lactic‐co‐glycolic acid) (PLGA) nanoparticles for oral administration: effect of polymer molecular weight and surface modification with chitosan on formulation, nanoparticle characterization and antibacterial effects. Polymers. 2019;11(10):1‐23. doi:10.3390/polym11101632 PMC6835525 31600969

[15] Villanueva MA , Torres RC , Baser KHC , Ozek T , Kurkcuoglu M . The composition of Manila elemi oil. Flavour Fragrance J. 1993;8:35‐37. doi:10.1002/ffj.2730080107

[16] Öztürk AA , Namlı İ , Güleç K , Görgülü Ş . Design of lamivudine loaded nanoparticles for oral application by nano spray drying method: a new approach to use an antiretroviral drug for lung cancer treatment. Comb Chem High Throughput Screen. 2020;23(10):1063‐1078. 10.2174/1386207323666200325155020 32209039

[17] Senel B , Öztürk AA . New approaches to tumor therapy with siRNA‐decorated and chitosan‐modified PLGA nanoparticles. Drug Dev Ind Pharm. 2019;45(11):1835‐1848. doi:10.1080/03639045.2019.1665061 31491363

[18] Öztürk AA , Namlı İ , Güleç K , Kıyan HT . Diclofenac sodium loaded PLGA nanoparticles for inflammatory diseases with high anti‐inflammatory properties at low dose: formulation, characterization and in vivo HET‐CAM analysis. Microvasc Res. 2020;130:103991. doi:10.1016/j.mvr.2020.103991 32105668

[19] Öztürk AA , Yenilmez E , Şenel B , Kıyan HT , Güven UM . Effect of different molecular weight PLGA on flurbiprofen nanoparticles: formulation, characterization, cytotoxicity and in vivo anti‐inflammatory effect by using HET‐CAM assay. Drug Dev Ind Pharm. 2020;46(4):682‐695. doi:10.1080/03639045.2020.1755304 32281428

[20] Dongmo PMJ , Tchoumbougnang F , Ndongson B , et al. Chemical characterization, antiradical, antioxidant and anti‐inflammatory potential of the essential oils of *Canarium schweinfurthii* and *Aucoumea klaineana* (Burseraceae) growing in Cameroon. Agric Biol J North Am. 2010;1(4):606‐611.

[21] Koudou J , Abena AA , Ngaissona P , Bessière JM . Chemical composition and pharmacological activity of essential oil of *Canarium schweinfurthii* . Fitoterapia. 2005;76(7–8):700‐703. doi:10.1016/j.fitote.2005.06.004 16239074

[22] Duymuş HG , Çiftçi GA , Yıldırım ŞU , Demirci B , Kırımer N . The cytotoxic activity of *Vitex agnus castus* L. essential oils and their biochemical mechanisms. Industrial Crops Prod. 2014;55:33‐42. doi:10.1016/j.indcrop.2014.01.041

[23] Mohamed K , Zine K , Fahima K , Abdelfattah E , Sharifudin SM , Duduku K . NiO nanoparticles induce cytotoxicity mediated through ROS generation and impairing the antioxidant defense in the human lung epithelial cells (A549): preventive effect of *Pistacia lentiscus* essential oil. Toxicol Rep. 2018;5:480‐488. doi:10.1016/j.toxrep.2018.03.012 29854619 PMC5977410

[24] Girola N , Figueiredo CR , Farias CF , et al. Camphene isolated from essential oil of *Piper cernuum* (Piperaceae) induces intrinsic apoptosis in melanoma cells and displays antitumor activity in vivo. Biochem Biophys Res Commun. 2015;467(4):928‐934. doi:10.1016/j.bbrc.2015.10.041 26471302

[25] Arunasree KM . Anti‐proliferative effects of carvacrol on a human metastatic breast cancer cell line, MDA‐MB 231. Phytomedicine. 2010;17(8–9):581‐588. doi:10.1016/j.phymed.2009.12.008 20096548

[26] Senthil Kumar KJ , Gokila Vani M , Wang C‐S , et al. Geranium and lemon essential oils and their active compounds downregulate angiotensin‐converting enzyme 2 (ACE2), a SARS‐CoV‐2 spike receptor‐binding domain, in epithelial cells. Plan Theory. 2020;9(6):770. doi:10.3390/plants9060770 PMC7355681 32575476

[27] Yu X , Lin H , Wang Y , et al. D‐limonene exhibits antitumor activity by inducing autophagy and apoptosis in lung cancer. Onco Targets Ther. 2018;11:1833‐1847. doi:10.2147/OTT.S155716 29670359 PMC5894671

[28] Zhang Z , Guo S , Liu X , Gao X . Synergistic antitumor effect of α‐pinene and β‐pinene with paclitaxel against non‐small‐cell lung carcinoma (NSCLC). Drug Res. 2014;65(4):214‐218. doi:10.1055/s-0034-1377025 25188609

[29] Servi H , Demir U , Servi EY , Gundogdu B , Barak TH . Antiproliferative and antibacterial activities of four Commer‐cial essential oil samples from Boswellia carteri, B. serrata, and two chemotypes of Canarium luzonicum. J. Essent. Oil Bear. Plants. 2023;26(1):79‐94.

